# Use of Anti-Diabetic Agents in Non-Diabetic Kidney Disease: From Bench to Bedside

**DOI:** 10.3390/life11050389

**Published:** 2021-04-25

**Authors:** Sungjin Chung, Gheun-Ho Kim

**Affiliations:** 1 Department of Internal Medicine, College of Medicine, The Catholic University of Korea, Seoul 06591, Korea; chungs@catholic.ac.kr; 2 Department of Internal Medicine, Hanyang University College of Medicine, Seoul 04763, Korea

**Keywords:** dipeptidyl peptidase-4 inhibitor, glucagon-like peptide-1 receptor agonist, inflammation, metformin, oxidative stress, sodium-glucose transporter-2 inhibitor

## Abstract

New drugs were recently developed to treat hyperglycemia in patients with type 2 diabetes mellitus (T2D). However, metformin remains the first-line anti-diabetic agent because of its cost-effectiveness. It has pleiotropic action that produces cardiovascular benefits, and it can be useful in diabetic nephropathy, although metformin-associated lactic acidosis is a hindrance to its use in patients with kidney failure. New anti-diabetic agents, including glucagon-like peptide-1 receptor (GLP-1R) agonists, dipeptidyl peptidase-4 (DPP-4) inhibitors, and sodium-glucose transporter-2 (SGLT-2) inhibitors, also produce cardiovascular or renal benefits in T2D patients. Their glucose-independent beneficial actions can lead to cardiorenal protection via hemodynamic stabilization and inflammatory modulation. Systemic hypertension is relieved by natriuresis and improved vascular dysfunction. Enhanced tubuloglomerular feedback can be restored by SGLT-2 inhibition, reducing glomerular hypertension. Patients with non-diabetic kidney disease might also benefit from those drugs because hypertension, proteinuria, oxidative stress, and inflammation are common factors in the progression of kidney disease, irrespective of the presence of diabetes. In various animal models of non-diabetic kidney disease, metformin, GLP-1R agonists, DPP-4 inhibitors, and SGLT-2 inhibitors were favorable to kidney morphology and function. They strikingly attenuated biomarkers of oxidative stress and inflammatory responses in diseased kidneys. However, whether those animal results translate to patients with non-diabetic kidney disease has yet to be evaluated. Considering the paucity of new agents to treat kidney disease and the minimal adverse effects of metformin, GLP-1R agonists, DPP-4 inhibitors, and SGLT-2 inhibitors, these anti-diabetic agents could be used in patients with non-diabetic kidney disease. This paper provides a rationale for clinical trials that apply metformin, GLP-1R agonists, DPP-4 inhibitors, and SGLT-2 inhibitors to non-diabetic kidney disease.

## 1. Introduction

Chronic kidney disease (CKD) is a major public health burden, affecting more than 750 million people worldwide [1]. Because of the increasing global prevalence of type 2 diabetes mellitus (T2D) in CKD patients, CKD can be classified into diabetic kidney disease (DKD) and non-diabetic CKD. DKD accounted for 47% of patients initiating kidney replacement therapy due to end stage kidney disease (ESKD) in the United States in 2018 [2], and 48% in South Korea in 2019 [3].

During the past decade, a series of new anti-diabetic agents has been developed and validated to lower glycemia. Those drugs also carry cardiovascular and renal benefits and risks for patients with T2D. Thiazolidinediones can cause fluid retention and an increased risk of heart failure in patients with T2D [4]. However, glucagon-like peptide-1 receptor (GLP-1R) agonists are associated with favorable cardiovascular [5] and renal [6] outcomes in patients with T2D. Dipeptidyl peptidase-4 (DPP-4) inhibitors carry neither risk nor benefit to the cardiovascular system [7]. Sodium-glucose transporter-2 (SGLT-2) inhibitors emerged as game changers because they brought absolute benefits to cardiovascular [8] and renal [9] outcomes in patients with T2D.

The beneficial effects of these anti-diabetic agents on the cardiovascular system are independent of their glucose-lowering action. In particular, proteinuria reduction can be achieved by systemic or glomerular hemodynamic stability and inflammatory modulation. Consistent with that, GLP-1R agonists and SGLT-2 inhibitors reduce blood pressure and can preserve kidney function. As seen in the action of angiotensin II in the kidney and vasculature [10], hypertension, proteinuria, and renal inflammation are still the most important mediators for renal progression in both DKD and non-diabetic CKD. In contrast with DKD, no remarkable agents have been identified as effective measures to treat non-diabetic kidney disease during the past decade. Recent clinical trials elucidated the ability of SGLT-2 inhibitors to treat heart failure [11] and CKD [12] in patients without diabetes mellitus. However, clinical data are lacking to demonstrate their efficacy in specific non-diabetic kidney diseases. This paper provides a rationale for conducting clinical trials to test the use of metformin, GLP-1R agonists, DPP-4 inhibitors, and SGLT-2 inhibitors in various non-diabetic kidney diseases.

## 2. Metformin

Metformin is a biguanide, a drug class of herbal origin that has been widely used since the 1950s, and it is currently the first-line pharmacologic treatment for T2D [13]. Metformin acts mainly in the liver, inhibiting gluconeogenesis by blocking mitochondrial redox transfer [14]. However, its effects are likely pleiotropic because metformin acts on the metabolism and inflammation via both adenosine monophosphate-activated protein kinase (AMPK)-dependent and AMPK-independent mechanisms, leading to kidney protection. Potential uses of metformin in kidney disease have been extensively reviewed elsewhere [15,16,17]. 

### 2.1. Acute Kidney Injury

Considering the risk of metformin-associated lactic acidosis, early guidelines mention (without evidence) the need to stop metformin before using intravascular contrast media [18,19]. However, multiple studies and meta-analyses have shown that the risk of lactic acidosis is very low and linked more to the underlying disease and co-morbidities than to the use of metformin [20]. Current guidelines say that patients with an estimated glomerular filtration rate (eGFR) > 45 mL/min/1.73 m^2^ can continue to take metformin before and after exposure to iodine-based contrast media [21,22]. Yu et al. recently reported that in diabetic patients with eGFR > 30 mL/min/1.73 m^2^, the continued use of metformin did not increase the risk of contrast-induced-acute kidney injury (AKI) after primary percutaneous coronary intervention for ST-segment elevation myocardial infarction [23]. In patients without diabetes or prior renal impairment, no adverse effect of metformin on renal function was reported after myocardial infarction and subsequent contrast exposure [24].

On the other hand, metformin offers protective effects against AKI in experimental animals. Li et al. reported that metformin protected against cisplatin-induced tubular cell apoptosis and AKI by stimulating AMPKα activation and autophagy induction in tubular cells [25]. In a rat model of renal ischemia/reperfusion injury, metformin markedly relieved insufficient autophagic flux in the kidney cortex and improved cellular stress and apoptotic markers [26]. Autophagy is the physiologic, regulated mechanism by which cells remove unnecessary or dysfunctional components [27]. Under pathological conditions, renal cells upregulate autophagy in response to cell stress, but maladaptive autophagy or insufficient autophagic flux can induce apoptosis and renal injury [28,29]. In short, metformin induces autophagy, which provides beneficial cellular effects.

Metformin also effectively blocked AKI in a rat model of gentamicin nephrotoxicity [30]. Gentamicin induces mitochondrial dysfunction, which produces reactive oxygen species (ROS) [31]. Potential therapeutic approaches to mitochondrial dysfunction in kidney disease include metformin and other drugs [32].

### 2.2. Chronic Kidney Disease 

Renal mass reduction and chronic adenine administration are two representative animal models of CKD. Metformin improved kidney function and ameliorated kidney fibrosis and structural alterations in an ablation and infarction rat model of subtotal nephrectomy [33]. Those authors concluded that restoring AMPK activity could suppress the progressive loss of renal function in non-diabetic CKD. In a rat model of adenine-induced CKD, metformin prevented renal progression and features of mineral and bone disorder such as hyperphosphatemia, hypocalcemia, secondary hyperparathyroidism, and vascular calcification [34]. As expected, metformin also ameliorated cellular infiltration, fibrosis, and inflammation in the kidney.

Unilateral ureteral obstruction (UUO) is an animal model frequently used to investigate renal interstitial fibrosis. Metformin attenuated kidney inflammation and fibrosis in mice with UUO [35]. Feng et al. found that metformin reduced UUO-induced transforming growth factor β1 (TGFβ1) mRNA and protein expression by stimulating AMPKα2-dependent targeting of TGFβ1 production and AMPKα2-independent targeting of Smad3 phosphorylation downstream of TGFβ1 [36]. In an AMPK-independent pathway, metformin inhibited the activation of ERK signaling and attenuated the production of extracellular matrix proteins and collagen deposition in the obstructed kidneys [37]. The DEP domain-containing mTOR interacting protein (DEPTOR) is an endogenous negative regulator of mTOR that inhibits the kinase activity of both mammalian target of rapamycin complex 1 (mTORC1) and mTORC2. According to Wang et al., metformin attenuated renal interstitial fibrosis by increasing DEPTOR expression and inhibiting the mTOR/p70S6K pathway in the kidneys of UUO rats [38]. Because metformin limited the infiltration of immune cells into the UUO kidney, systemic immunomodulatory action was suggested, probably via inhibition of signal transducer and activator of transcription 3 (STAT3) activity [39]. Systemic lupus erythematosus is an autoimmune disease, and immune cells can be therapeutic targets of metformin. In *Roquin^san/san^* mice, metformin attenuated inflammation in kidney and liver tissues and inhibited B cell differentiation into plasma cells and the formation of germinal centers in association with enhanced AMPK expression and the inhibition of mTOR-STAT3 signaling [40].

Proteinuria plays an important role in the pathogenesis of CKD, and it can be modified by metformin. In spontaneously hypertensive rats, metformin reduced proteinuria and increased the production of vascular endothelial growth factor (VEGF)-A in rat kidneys, probably by hypoxia-inducible factor (HIF)-2α activation [41]. A cell experiment mimicking albuminuria explored the beneficial action mechanisms of metformin. Metformin treatment restored AMPK phosphorylation and augmented autophagy in rat renal proximal tubular (NRK-52E) cells exposed to albumin. In addition, metformin treatment attenuated the albumin-induced phosphorylation of protein kinase B (AKT) and the downstream targets of mTOR and prevented the albumin-mediated induction of epithelial-mesenchymal transition marker α-SMA, pro-apoptotic endoplasmic reticulum (ER) stress marker CHOP, and apoptotic caspases -12 and -3 in renal cells [42]. 

In clinical practice, however, metformin has not been used for non-diabetic kidney diseases. A phase 3 randomized controlled trial (Metformin as RenoProtector of Progressive Kidney Disease (RenoMet); NCT03831464) is ongoing to test the effects of metformin in stage 2 and 3 CKD [43]. Table 1 summarizes the results of metformin treatment in animal models of non-diabetic kidney disease.

### 2.3. Autosomal Dominant Polycystic Kidney Disease

Autosomal Dominant Polycystic Kidney Disease (ADPKD) is the most common hereditary kidney disease, and it frequently leads to ESKD during or after the sixth decade of life through the development and inexorable expansion of multiple cysts throughout the kidney parenchyma [44]. Although hypertension control is important to protect kidney function, disease-modifying therapeutic drugs based on ADPKD pathophysiology are needed. An increase in cyclic adenosine monophosphate (cAMP) plays an important role in generating and maintaining fluid-filled cysts in collecting duct principal cells because cAMP activates protein kinase A (PKA) and stimulates epithelial chloride secretion through the cystic fibrosis transmembrane conductance regulator (CFTR) [45]. A vasopressin V2 receptor antagonist, tolvaptan, was recently found to successfully preserve kidney function in ADPKD by targeting the role of vasopressin-mediated cAMP in cyst growth [44].

**Figure 1:**
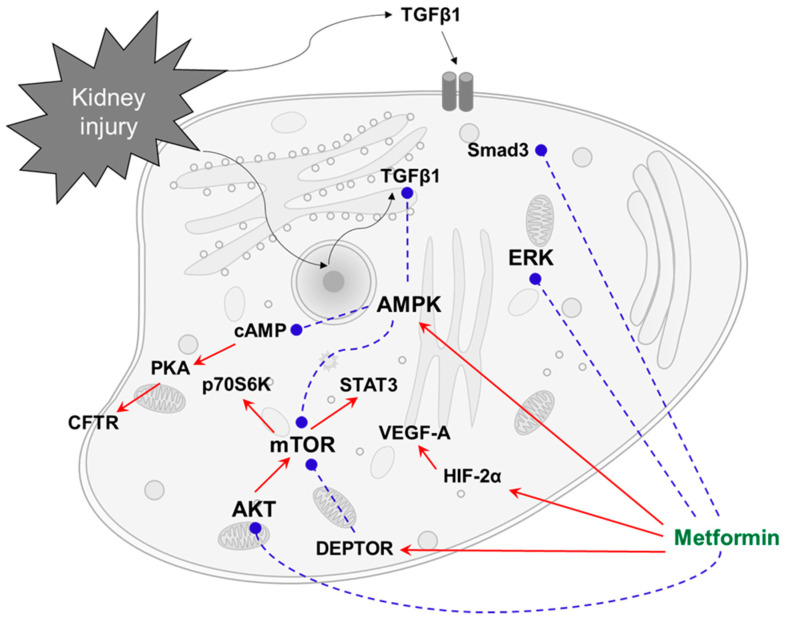
Intracellular pathways for the action of metformin that lead to renoprotection in non-diabetic kidney disease. AMPK activation inhibits TGFβ1 and mTOR and acts against inflammation and cell death. cAMP suppression could inactivate PKA and CFTR in ADPKD. AMPK-independent pathways include the inhibition of ERK and AKT signaling, which acts against cell proliferation and apoptosis. mTOR inhibition via DEPTOR can also improve autophagic flux. Red arrows indicate stimulation, and blue broken lines indicate inhibition. AMPK, 5’ adenosine monophosphate-activated protein kinase; AKT, protein kinase B; cAMP, cyclic adenosine monophosphate; CFTR, cystic fibrosis transmembrane conductance regulator; DEPTOR, DEP domain-containing mTOR-interacting protein; ERK, extracellular signal-regulated kinase; HIF-2α, hypoxia-inducible factor-2α; mTOR, mammalian target of rapamycin; PKA, protein kinase A; p-Smad3, phosphorylated mothers against decapentaplegic homolog 3; PKA, protein kinase A; STAT3, signal transducer and activator of transcription 3; TGFβ1, transforming growth factor β1; VEGF-A, vascular endothelial growth factor-A.

Metformin suppresses glucagon-dependent glucose output from hepatocytes by reducing cAMP production and PKA activity via AMPK activation [46], which inhibits the CFTR chloride channel activity in polarized epithelia [47]. Consistently, metformin was shown to slow renal cystogenesis *in vitro* and *ex vivo* and to produce a significant decrease in cystic growth in two different mouse models of ADPKD [48]. The intracellular pathways of metformin action for non-diabetic kidney diseases are summarized in Figure 1.

Pisani et al. retrospectively compared the decline in eGFR between seven diabetic ADPKD patients treated with metformin and seven matched non-diabetic ADPKD controls not receiving metformin treatment [49]. During three years of follow-up, they found that renal progression was slower when metformin was used. A phase II randomized placebo-controlled clinical trial completed on 7 December 2020, assessed the safety, tolerability, and effects of metformin treatment on kidney volume growth and eGFR in patients with early to moderate ADPKD (eGFR ≥ 50 mL/min/1.73 m^2^) [50]. The results from another clinical trial (NCT02903511) testing the feasibility of metformin therapy in ADPKD are being analyzed.

It is well-known that metformin can cause subclinical increases in lactic acid and lactic acidosis in extreme overdose, but long-term experience and trial data have shown no safety concerns for metformin use except in a relatively small subset of patients with severe liver, heart, or renal dysfunction [14]. Ongoing trials will address the safety concerns for metformin use in non-diabetic kidney diseases.

## 3. Glucagon-Like Peptide-1 Receptor Agonists

**Figure 2:**
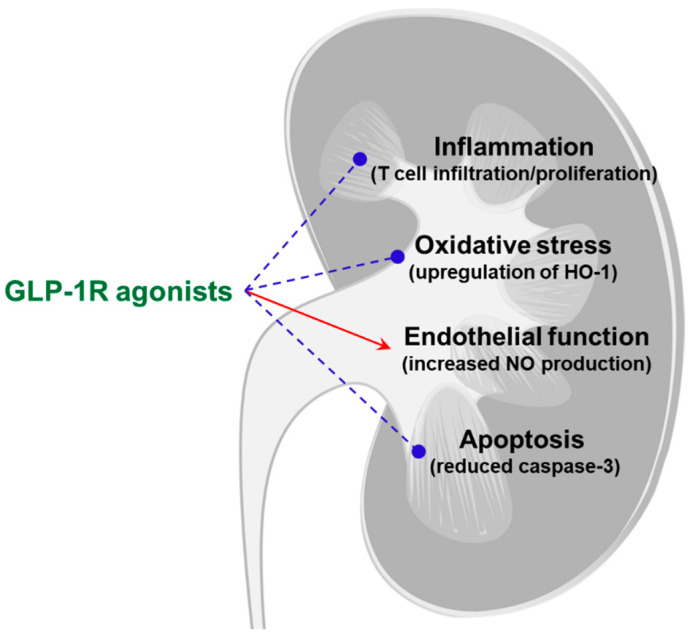
Potential mechanisms of renoprotection induced by GLP-1R agonists in non-diabetic kidney disease. The red arrow indicates stimulation, and blue broken lines indicate inhibition. GLP-1R, glucagon-like peptide-1 receptor; HO-1, heme oxyagenase-1; NO, nitric oxide.

In response to food intake, glucagon-like peptide-1 (GLP-1) is secreted by intestinal endocrine cells to facilitate insulin secretion from pancreatic β-cells. GLP-1 exerts its effects by binding to GLP-1R and subsequently activating adenylate cyclase, which leads to the generation of cAMP. cAMP stimulates insulin secretion by activating PKA and exchange factor directly activated by cAMP 2 (EPAC2) in pancreatic β-cells [51]. The glucose-lowering effect of GLP-1 occurs via stimulation of glucose-dependent release of insulin from pancreatic islet cells, slowing gastric emptying and decreasing appetite stimulation in the brain. This is the pathway by which GLP-1R agonists could improve blood glucose and confer weight loss [51,52]. In addition to the pancreas, GLP-1R is expressed in multiple organs, such as the gut, kidneys, heart, and central nervous system [52]. Thus, the GLP-1R agonists can exert extrapancreatic action to protect multiple organs in the body, including the cardiovascular system, lungs, and kidneys [53]. The beneficial effects of GLP-1 on the cardiovascular system, such as blood pressure control and improvement of endothelial function, can also extend the renal protection (Figure 2). In particular, the GLP-1R agonists have anti-apoptotic and anti-inflammatory action and can increase nitric oxide production [54]. 

### 3.1. Acute Kidney Injury

Contrast-induced nephropathy, the major cause of hospital-acquired AKI, has multiple pathophysiology mechanisms, including renal hypoxia, oxidative stress, and endothelial dysfunction [55]. Hussien et al. showed the prophylactic effect of exendin-4, a GLP-1R agonist, against contrast-induced nephropathy in a rat model. Pretreatment with exendin-4 ameliorated biomarkers of renal function, oxidative stress, vascular dysfunction, and apoptosis [56]. Similar results were obtained from a rat model of renal ischemia/reperfusion injury. When the rats were pretreated with exendin-4 before reperfusion, the kidney injury was attenuated by reducing the expression of caspase-3 and macrophage infiltration and increasing heme oxygenase-1 (HO-1) expression [57]. Exendin-4 also reduced cisplatin-induced renal injury and apoptosis in mice [58].

### 3.2. Chronic Kidney Disease

Few data are available from the use of GLP-1R agonists in non-diabetic CKD. The anti-inflammatory action of an GLP-1R agonist (liraglutide) was shown in a mouse model of T cell–mediated glomerulonephritis [59]. Liraglutide treatment decreased renal infiltration and the proliferation of T cells, but albuminuria was not improved. Table 2 summarizes the treatment results of GLP-1R agonists in animal models of non-diabetic kidney disease.

An arteriovenous fistula is the vascular access required to maintain hemodialysis in ESKD patients. Thrombotic tendency is inevitable due to the turbulent blood flow and endothelial damage. Chien et al. investigated whether exendin-4 could relieve arteriovenous fistula injury in rats with CKD [60]. They reported that exendin-4 treatment restored normal endothelial morphology and improved arteriovenous fistula function by upregulating HO-1, consistent with the role of hypoxia and oxidative stress in immature arteriovenous fistulae [61,62].

Since the overall safety data for GLP-1R agonists from previous clinical trials in diabetic patients are reassuring, a few side effects of GLP-1R agonists including gastrointestinal symptoms, injection-site reactions, and an increased heart rate can preclude use of GLP-1R agonists in some cases, and such use needs to be avoided in subjects with medullary thyroid tumor or history of acute pancreatitis [63].

## 4. Dipeptidyl Peptidase-4 Inhibitors

The pharmacologic action of DPP-4 inhibitors is similar to that of GLP-1R agonists. The major therapeutic effects of DPP-4 inhibitors protect against degradation of the substrates GLP-1 and glucose-dependent insulinotropic polypeptide (GIP), which are physiological substrates that affect insulin and glucagon secretion in a glucose-dependent manner [64]. GLP-1 accumulates with the inhibition of DPP-4 because the soluble form of DPP-4 circulates in the plasma and rapidly degrades GLP-1. However, DPP-4 is also expressed as a membrane-bound form in a variety of tissues, primarily on endothelial and epithelial cells [65]. In the kidney, DPP-4 is expressed on the brush border of the proximal tubules and glomerular podocytes [66].

**Figure 3:**
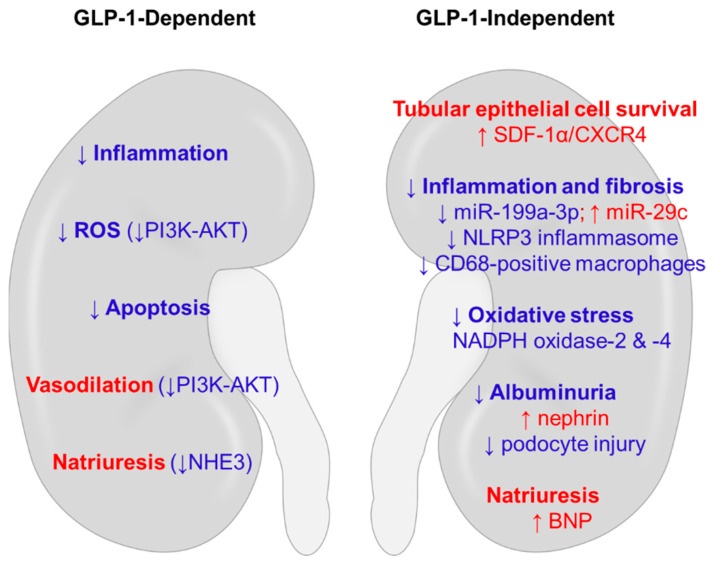
Potential mechanisms of renoprotection induced by DPP-4 inhibitors in non-diabetic kidney disease. The anti-inflammatory, anti-oxidative, and anti-apoptotic actions of DPP-4 inhibitors use both GLP-1-dependent and GLP-1-independent mechanisms. Hemodynamic benefits could be conferred through natriuresis and vasodilation. Red words denote stimulatory effects, and blue words denote inhibitory effects. BNP, brain-derived natriuretic peptide; CXCR4, C-X-C chemokine receptor type 4; DPP-4, dipeptidyl peptidase-4; GLP-1, glucagon-like peptide-1; NADPH, nicotinamide adenine dinucleotide phosphate; NHE3, Na^+^/H^+^ exchanger type 3; NLRP3, NLR family pyrin domain containing 3; PI3K-AKT, phosphatidylinositol 3-kinase-protein kinase B; SDF-1α, stromal-derived factor-1α.

In addition to the extrapancreatic action derived from GLP-1, DPP-4 inhibitors can offer organ protection via GLP-1-independent mechanisms [53]. The enzyme DPP-4 cleaves multiple peptides other than GLP-1, such as brain-derived natriuretic peptide (BNP), neuropeptide Y (NPY), and stromal-derived factor (SDF)-1α. Thus, multiple substrates might be responsible for the pleiotropic action of DPP-4 inhibitors (Figure 3).

### 4.1. Acute Kidney Injury

Vildagliptin pre-treatment in a rat model of ischemia/reperfusion injury preserved kidney function in association with reduced tubular necrosis and decreased apoptotic, oxidative, and proinflammatory markers [67]. Post-treatment with sitagliptin offered similar benefits in terms of kidney recovery and pleiotropic actions after acute ischemia/reperfusion injury [68].

Treatment with alogliptin reduced cisplatin–induced AKI and reduced the renal mRNA expression ratios of Bax/Bcl-2 and Bim/Bcl-2, markers of apoptosis [58]. In addition, the cisplatin-induced increase in the levels of other DPP-4 substrates, such as SDF-1α and NPY, was reversed. Teneligliptin also attenuated cisplatin-induced AKI and accelerated kidney recovery by promoting the proliferation of surviving epithelial cells in the proximal tubule via the chemokine ligand CXCL12 (or SDF-1α) and its receptor chemokine receptor 4 (CXCR4) [69]. Upregulation of the mRNA expression of both SDF-1α and CXCR4 was also found in the kidney after acute ischemia/reperfusion injury [70]. Thus, the SDF-1α/CXR4 axis could have a role in kidney repair by regenerating tubular epithelial cells in both ischemic and nephrotoxic injury. As shown in Figure 3, SDF-1α is an important DPP-4 substrate that potentially mediates the protective effects of DPP-4 inhibition in the kidney.

Natriuresis induced by DPP-4 inhibitors or GLP-1R agonists could be linked to renoprotection (Figure 3). Active sodium transport along the nephron is primarily driven by basolaterally located Na^+^-K^+^-ATPase that uses ATP hydrolysis as a source of energy. That process requires oxygen consumption to maintain a sustained rate of ATP generation in the kidney [71]. Na^+^/H^+^ exchanger type 3 (NHE3) is the major sodium transporter in the proximal tubule, and Girardi et al. reported that the administration of a DPP-4 inhibitor to Wistar rats for 7 days reduced both NHE3 activity and protein abundance in the proximal tubule brush border [72]. They also reported that NHE3 activity in LLC-PK1 cells was decreased by treatment with exendin-4 [73]. Downregulation of NHE3 could limit energy consumption in the proximal tubule and protect the kidney from acute ischemia/reperfusion injury [74].

### 4.2. Chronic Kidney Disease

As in diabetic nephropathy, albuminuria is an important marker of CKD and indicator of renal disease progression. Although DPP-4 inhibition appears to effectively ameliorate albuminuria [7], it is unlikely to improve renal survival in T2D patients [75]. It should be determined whether DPP-4 inhibitors are useful in patients with non-diabetic kidney disease. 

Many preclinical studies have shown the renoprotective effects of DPP-4 inhibition in non-diabetic CKD. Alogliptin treatment ameliorated renal inflammation and fibrosis in mice with UUO [76]. Evogliptin also attenuated UUO-induced renal atrophy and tubulointerstitial fibrosis in association with the inhibition of pro-fibrotic gene expression and extracellular matrix production [77]. Consistent with that, linagliptin suppressed the induction of pro-fibrotic miRNA such as miR-199a-3p and restored levels of the anti-fibrotic miR-29c in rats with 5/6 nephrectomy [78]. Linagliptin also reduced albuminuria and attenuated glomerular hypertrophy and interstitial fibrosis in non-diabetic rats with 5/6 nephrectomy [79]. Joo et al. showed that in the rat remnant kidney model, sitagliptin improved renal functional and morphological changes by attenuating activation of the phosphatidylinositol 3-kinase (PI3K)-AKT pathway [80]. In aging mice, linagliptin improved kidney function and tubulointerstitial fibrosis in association with alterations to nicotinamide adenine dinucleotide phosphate (NADPH) oxidase-2 and NADPH oxidase-4 [81].

The anti-inflammatory action of DPP-4 inhibition has also been shown in animal models of glomerulopathy. Alogliptin reduced the number of CD68-positive inflammatory macrophages in the kidney in a rat Thy-1 glomerulonephritis model [82]. Linagliptin pre-treatment in anti-GBM nephritic rats reduced the number of crescents, glomerulosclerosis, tubular injury, and renal fibrosis [83]. In mice with doxorubicin nephropathy, evogliptin reduced albuminuria in association with restored nephrin expression in podocytes and decreased podocyte injury [84]. Sitagliptin and linagliptin ameliorated NLRP3 inflammasome activation and oxidative stress markers in rats with doxorubicin nephropathy [85].

The anti-inflammatory action of DPP-4 inhibition was also demonstrated in animal models of salt-sensitive hypertension. Vildagliptin attenuated the development of salt-induced hypertension in Dahl salt-sensitive rats by increasing urine sodium excretion [86]. In addition, sitagliptin improved albuminuria and serum creatinine in Dahl salt-sensitive rats in association with the amelioration of inflammatory markers in the kidney [87]. Saxagliptin also improved albuminuria and suppressed inflammation- and fibrosis-related genes in Dahl salt-sensitive rats [88]. Table 3 summarizes the results of DPP-4 inhibitor treatment in animal models of non-diabetic kidney disease.

Although a few potential risks associated with DPP-4 inhibitors have been reported with respect to effects in the immune system and risk of acute pancreatitis, there is a relative lack of unwanted off-target or adverse effects associated with the DPP-4 inhibitors that are used therapeutically [64].

## 5. Sodium-Glucose Transporter-2 Inhibitors

The kidney plays an important role in glucose homeostasis via gluconeogenesis, glucose utilization, and glucose reabsorption from the glomerular filtrate [89]. Glucose is produced in the renal cortex and contributes 20% to 25% of the total body glucose in the fasting state. Glucose is also used in the renal medulla, accounting for 10% of total glucose uptake by the body in the fasting state [90]. In addition, plasma glucose is freely filtered through glomeruli and completely reabsorbed in the proximal tubule via apical SGLTs and basolateral glucose transporters (GLUTs). In the early proximal tubule, SGLT-2 acts as a high-capacity, low-affinity (sodium: glucose ratio of 1:1) secondary active transporter and is responsible for most of the renal glucose reabsorption, in concert with GLUT-2. The remaining glucose is reabsorbed by SGLT-1 (sodium: glucose ratio of 2:1) in the late proximal tubule and then reabsorbed into the blood via GLUT-1 [91].

In patients with T2D, renal glucose metabolism is altered. Both renal and hepatic gluconeogenesis are increased in the fasting state [92]. Postprandial renal glucose release also increases to a greater extent in patients with T2D than in people with normal glucose tolerance [93]. In parallel with increased glucose production, renal glucose uptake or utilization is increased in both the fasting and postprandial states in patients with T2D. Renal tubular transport physiology is also altered in diabetic patients. Glucose reabsorption is increased in the proximal tubule in response to the increased filtered load of glucose. Specifically, the transport maximum for glucose is increased by the upregulation of SGLT-2 in the proximal tubule [94]. Thus, diabetic patients can have glucosuria at higher-than-normal plasma glucose levels [91]. 

The upregulation of SGLT-2 in patients with T2D is associated with important metabolic and hemodynamic consequences because multiple therapeutic benefits are produced by SGLT-2 inhibition [95]. First, SGLT-2 inhibitors increase urinary glucose and calorie excretion, thereby reducing plasma glucose levels and body weight. Second, the accompanying natriuresis induced by SGLT-2 inhibition lowers blood pressure and diabetic glomerular hyperfiltration. Initially, glomerular hypertrophy and hyperfiltration are induced by hyperglycemia in diabetic patients. However, they can be aggravated by altered tubuloglomerular feedback at later stages. The upregulation of SGLT-2 in the proximal tubule decreases NaCl delivery to the macula densa, where adenosine is released to regulate preglomerular afferent arteriolar resistance. Thus, the decrease in adenosine release in response to reduced distal delivery of NaCl decreases afferent arteriolar resistance, leading to glomerular hyperfiltration [96]. This process can be reversed by SGLT-2 inhibition, reducing albuminuria and protecting the kidney.

The glomerular hyperfiltration theory is not limited to diabetic nephropathy; it could also be applicable to non-diabetic kidney disease [97]. The single-nephron glomerular filtration rate would be increased in response to a reduction in functioning renal mass in CKD, and it might be modulated by SGLT-2 inhibition, reducing albuminuria. Because hypertension in CKD is mainly salt-sensitive, it might also be responsive to SGLT-2 inhibition. Furthermore, oxidative stress and inflammatory activation are common pathways leading to both renal and cardiac fibrosis [98], and the cardiovascular benefits of SGLT-2 inhibition could be extended to favorable renal outcomes because of the heart–kidney connection.

Furthermore, SGLT-2 inhibitors could protect the kidney by reducing cortical hypoxia, in association with the downregulation of NHE3 in the proximal tubule [99]. In general, hypoxia induces cell stress, producing ROS and triggering ER stress from the accumulation of unfolded proteins in the ER [100]. Cells respond to ER stress by activating a series of integrative stress pathways, but if ER stress is chronic or excessive, the unfolded protein response becomes maladaptive and can become cytotoxic by activating apoptosis [101]. Mitochondrial dysfunction is also an important component of various kidney diseases, inducing ER stress and subsequent cellular damage [32]. Further ROS generation and the activation of pro-inflammatory pathways are promoted, although those cellular stresses and organellar derangements are normally constrained by a cellular housekeeping pathway known as autophagy, a lysosomally mediated degradative pathway that maintains cellular homeostasis in the kidney [102].

**Figure 4:**
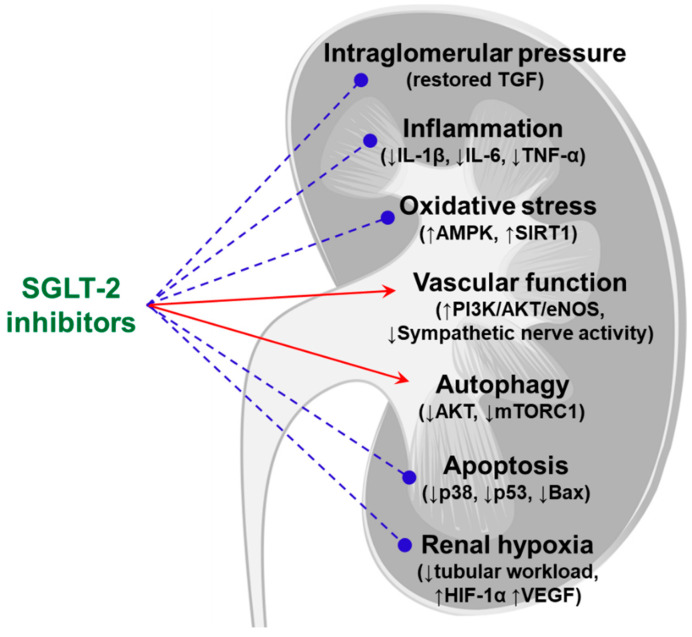
Potential mechanisms of renoprotection induced by SGLT-2 inhibitors in non-diabetic kidney disease. Pleiotropic effects include anti-inflammatory, anti-oxidative, and anti-apoptotic action by SGLT-2 inhibitors. Restoration of enhanced tubuloglomerular feedback could reduce albuminuria. Stimulation of the PI3K/AKT/eNOS pathway and inhibition of sympathetic nerve activity improve vascular endothelial function. SGLT-2 inhibition could affect NHE3 activity in the proximal tubule, reducing tubular workload. Simultaneously, the increase in VEGF induced by HIF-1α activation relieves renal hypoxia. Red arrows indicate stimulation, and blue broken lines indicate inhibition. AKT, protein kinase B; AMPK, 5’ adenosine monophosphate-activated protein kinase; Bax, bcl-2-like protein 4; eNOS, endothelial nitric oxide synthase; HIF-1α, hypoxia-inducible factor 1α; IL-1β, interleukin-lβ; IL-6, interleukin-6; mTORC1, mammalian target of rapamycin complex 1; NHE3, Na^+^/H^+^ exchanger type 3; PI3K, phosphoinositide 3-kinase; SGLT-2, sodium-glucose transporter-2; SIRT1, sirtuin-1; TNF-α, tumor necrosis factor-α; TGF, tubuloglomerular feedback; VEGF, vascular endothelial growth factor.

Figure 4 summarizes potential mechanisms for the renoprotection induced by SGLT-2 inhibition in non-diabetic kidney disease. Several cell-signaling pathways are involved in its pleiotropic action and lead to cardiovascular benefits. First, activation of the PI3K/AKT pathway decreases ROS generation and increases the phosphorylation of AKT/eNOS, which reduces inflammation and increases NO production [103]. Li et al. reported that phlorizin suppressed the expression of SGLT-1 and SGLT-2, activated the PI3K/AKT/eNOS signaling pathway, and increased the output of NO in palmitic acid–incubated human vascular endothelial cells [104]. Second, SGLT-2 inhibitors induce both AMPK and sirtuin-1, directly muting oxidative stress and inflammation and also stimulating autophagy to relieve cellular stress and renal injury [105]. Third, the SGLT-2 inhibitors might suppress the AKT and mTORC1 cell signaling pathway, ameliorating oxidative stress and inflammation and recovering autophagy [106]. Jaikumkao et al. recently reported that dapagliflozin ameliorated pancreatic oxidative stress, ER stress, inflammation, and apoptosis and restored kidney autophagy in obese rats [107].

### 5.1. Acute Kidney Injury

The possibility of provoking AKI might be a concern with SGLT-2 inhibitors because they produce a transient decrease in intraglomerular pressure. A total of 511 AKI events were reported among 36,716 T2D patients in 53 clinical trials [108]. However, SGLT-2 inhibitors reduced the risk of AKI by 25% when data were analyzed from the major randomized controlled trials [109].

The SGLT-2 inhibitors can protect kidneys from ischemic injury through several mechanisms. In the proximal tubule, SGLT-2 inhibition can reduce the accumulation of intracellular glucose and sodium and decrease the activity of Na^+^-K^+^-ATPase. Interestingly, NHE3 activity in the proximal tubule can be affected by SGLT-2 inhibition [110]. The resultant decrease in tubular workload contributes to reduced tissue oxygen consumption, which improves renal cortical hypoxia [111]. In an ischemia/reperfusion mouse model, dapagliflozin reduced renal damage in association with increased renal expression of HIF-1 [112]. HIF-1α stimulates erythropoiesis and upregulates the expression of VEGF, reducing systemic hypoxia and renal ischemia. Zhang et al. reported that luseogliflozin prevented endothelial rarefaction and subsequent renal fibrosis after renal ischemia/reperfusion injury in non-diabetic mice through a VEGF-dependent pathway [113].

The SGLT-2 inhibitors can protect kidneys by activating cell survival pathways. Canagliflozin attenuated cisplatin-induced nephropathy in C57BL/6 mice and suppressed cisplatin-induced renal proximal tubular cell apoptosis in association with the inhibition of p53, p38, and JNK activation in vitro [114]. Chang et al. also showed that dapagliflozin reduced renal expression of Bax, renal tubule injury, and TUNEL-positive cells in ischemia/reperfusion-injured mice and increased the expression of AMPK and ERK in hypoxic HK2 cells in vitro [112].

### 5.2. Chronic Kidney Disease

In the DAPA-CKD trial, the composite risk of a sustained decline in eGFR ≥ 50%, ESKD, or death from renal or cardiovascular causes was reduced by dapagliflozin treatment in CKD patients, regardless of the presence or absence of diabetes [12]. When the ongoing EMPA-Kidney trial is successfully completed, the indication for SGLT-2 inhibitors might be extended to non-diabetic CKD. However, whether the long-term use of SGLT-2 inhibitors will reduce proteinuria and preserve GFR is unclear at this time of writing [115].

Different animal models of non-diabetic kidney disease have shown conflicting results from SGLT-2 inhibition. The animal model of renal mass reduction could be appropriate to test the intact nephron hypothesis or the role of nephron loss in the progression of renal failure [116]. Zhang et al. administered dapagliflozin to 5/6 nephrectomized rats, but they found no attenuation of glomerulosclerosis or tubulointerstitial fibrosis and no changes in the overexpression of TGFβ1 mRNA [117]. A similar negative result was obtained using another selective SGLT-2 inhibitor, TA-1887, in 5/6 nephrectomized rats [118]. When empagliflozin was administered to C57BL/6N mice fed an oxalate-rich diet to induce chronic oxalosis, kidney dysfunction and markers of tubulointerstitial injury and fibrosis remained unchanged [119].

In rats with adenine-induced CKD, however, canagliflozin reduced albuminuria and plasma cystatin C, interleukin-1β, interleukin-6, and tumor necrosis factor-α [120]. Ipragliflozin also relieved plasma creatinine and interleukin-6 and ameliorated tubulointerstitial injury and 8-hydroxy-2′-deoxyguanosine expression in the kidneys of non-diabetic mice treated with adenine for 4 weeks [121]. Cassis et al. induced a mouse model of proteinuric non-diabetic nephropathy by administering bovine serum albumin after unilateral nephrectomy and found that dapagliflozin relieved proteinuria, glomerular lesions, and podocyte dysfunction and loss [122]. However, short-term treatment with dapagliflozin did not modify renal hemodynamic function or attenuate proteinuria in humans or in experimental focal segmental glomerulosclerosis [123]. Thus, whether SGLT-2 inhibition is renoprotective in the absence of hyperglycemia or SGLT-2 upregulation still needs to be determined.

In CKD animals, SGLT-2 inhibition had beneficial effects on salt-sensitive hypertension. We showed that the salt-sensitive hypertension induced by feeding uninephrectomized rats an 8% NaCl diet was controlled by empagliflozin treatment [124]. The lower blood pressure was not accompanied by natriuresis but was associated with increased renal expression of HIF-1α and the amelioration of renal inflammation. The recent DAPASALT trial also reported that dapagliflozin produced no significant natriuresis in association with its blood pressure lowering effect [125], which is compatible with the roles of inflammation, oxidative stress, and vascular dysfunction in hypertension [126]. In a rat model of angiotensin II-dependent hypertension, on the other hand, empagliflozin did not affect blood pressure but did prevent the development of renal glomerulosclerosis, tubulointerstitial fibrosis, an increase in inflammatory infiltrates, and the expression of collagen types I and IV [127]. Those results stress the anti-inflammatory action of SGLT-2 inhibitors, rather than their hemodynamic effects on the kidney. The salt-sensitive hypertension induced by feeding adenine-treated uninephrectomized rats an 8% NaCl diet was relieved by luseogliflozin treatment in association with a reduction in a low frequency of systolic arterial pressure, which reflects sympathetic nerve activity [128]. CKD is characterized by sympathetic hyperactivity, and SGLT-2 inhibitors might reduce sympathetic activation at the renal level [129].

Finally, renal hypoxia has a pathogenic role in both diabetic and non-diabetic kidney disease. In diabetes mellitus, renal perfusion for oxygen delivery can be reduced by hyperglycemia-associated microvascular injury. In addition, oxygen demand can be increased by the upregulation of sodium transport in the proximal tubule [130]. A mismatch between renal oxygen demand and oxygen delivery can occur in non-diabetic kidney disease as well. According to the intact nephron hypothesis, glomerular hyperfiltration and increasing tubular workload are inevitable in the remaining nephrons. A series of microvascular or endothelial injuries in CKD, irrespective of etiology, can produce vascular rarefaction and lead to tubulointerstitial inflammation and fibrosis [131]. Renal cortical hypoxia in CKD patients was demonstrated using blood oxygen level-dependent (BOLD)-magnetic resonance imaging (MRI) and was related to the aggravation of kidney function [132]. However, a recent clinical trial reported that in non-diabetic normotensive subjects, cortical or medullary tissue oxygenation examined using renal BOLD-MRI was unchanged by SGLT-2 inhibition [133]. Table 4 summarizes treatment results from SGLT-2 inhibitors in animal models of non-diabetic kidney disease.

A few risks of diabetic ketoacidosis, genital mycotic infections, and lower-limb amputations with SGLT-2 inhibitors have been reported in subjects with T2D [63]. However, it remains unclear whether these possible damages can occur in non-diabetic patients.

## 6. Conclusions

The glucose lowering–independent pleiotropic action of metformin, GLP-1R agonists, DPP-4 inhibitors, and SGLT-2 inhibitors might extend their indications to non-diabetic diseases. Preclinical studies have shown that all these anti-diabetic agents have robust anti-inflammatory and anti-oxidative action, leading to improvements in endothelial dysfunction. In addition, metformin could protect the kidney from acute or chronic injury by inhibiting apoptosis and inducing autophagy. Its effect on proteinuria and GFR will be verified by ongoing clinical trials in moderately advanced CKD patients. GLP-1R agonists and DPP-4 inhibitors could induce natriuresis and relieve hypertension via NHE3 downregulation or increased BNP release. Clinical evidence is required, although the beneficial effects of GLP-1R agonists and DPP-4 inhibitors on renal function have been reported in many preclinical studies. SGLT-2 inhibitors are emerging as a promising treatment for non-diabetic kidney disease. They can restore both systemic and glomerular hemodynamic alterations, leading to cardiorenal protection. Non-hemodynamic mechanisms include the relief of renal hypoxia, improvement of organellar dysfunction, inhibition of apoptosis, and induction of autophagy. Clinical trials are required to test whether the hemodynamic and non-hemodynamic mechanisms are connected to renal benefits in patients with non-diabetic kidney disease and without SGLT-2 upregulation.

## Figures and Tables

**Table 1:** Animal studies of metformin treatment for non-diabetic kidney disease.

Animal Model	Renal Function	Kidney Biomarker	Reference
Cisplatin-induced AKI (CD1 mice)	↓BUN	↓Tubular injury ↓Inflammatory cell infiltration ↑AMPKα activation ↑Autophagy ↓Apoptosis	[25]
Ischemia/reperfusion AKI (Wistar rats)	N/A	↓Tubulointerstitial damage ↓Oxidative stress ↓Pro-fibrotic markers ↑AMPK activity ↓mTOR activity ↑Autophagy ↓Apoptosis	[26]
Gentamicin-induced AKI (male rats)	↑GFR ↑RBF ↑RPF ↓RVR	↓Tubular necrosis ↓Oxidative stress ↓Mitochondrial dysfunction	[30]
Ablation/infarction CKD (Wistar rats)	↑GFR ↑RBF ↓Renal oxygen consumption per sodium reabsorbed	↑AMPK activity ↓Glomerulosclerosis ↓Interstitial fibrosis	[33]
Adenine-induced CKD (Wistar rats)	↓Serum creatinine ↑Creatinine clearance ↓Serum phosphorus	↓Tubulointerstitial injury ↓2,8-dihydroxyadenine crystals ↓Pro-inflammatory cytokines	[34]
UUO (C57BL/6 mice)	N/A	↑AMPKα activity ↓Pro-inflammatory cytokines ↓Macrophage infiltration ↓TGFβ1 and pSmad3, ↓Collagen I and α-SMA ↓Interstitial fibrosis	[35,36]
UUO (C57BL/6J mice)	N/A	↓ERK activation ↓TGFβ ↓Fibronectin and collagen I ↓Interstitial fibrosis	[37]
UUO (Sprague-Dawley rats)	↓BUN ↓Serum creatinine	↓Macrophage infiltration ↓Interstitial fibrosis ↑DEPTOR ↓mTOR-p70S6K	[38]
UUO (C57BL/6NRj mice)	N/A	↓KIM-1, α-SMA and HMGB1 ↓Immune cell infiltration ↓pSTAT3 ↓CXCL2/MIP-2 and CXCL1/KC	[39]
Lupus nephritis (*Roquin^san/san^* mice)	N/A	↓Nephritis histopathology ↑AMPK ↓mTOR-STAT3	[40]
Hypertensive nephropathy (SHR)	↓Proteinuria ↓Serum creatinine	↓Podocyte foot process effacement ↑VEGF-A and HIF-2α	[41]

Note: AKI, acute kidney injury; AMPK, adenosine monophosphate-activated protein kinase; α-SMA, α-smooth muscle actin; BUN, blood urea nitrogen; CKD, chronic kidney disease; CXCL, chemokine ligand; DEPTOR, DEP domain-containing mTOR interacting protein; GFR, glomerular filtration rate; HIF-2α, hypoxia inducible factor-2α; HMGB1, high mobility group box protein 1; KC, keratinocyte-derived chemokine; KIM-1, kidney injury molecule-1; MIP-2, macrophage inflammatory protein-2; mTOR, mammalian target of rapamycin; N/A, not available; pSmad3, phospho-Smad3; pSTAT3, phospho-signal transducer and activator of transcription 3; p70S6K, ribosomal protein S6 kinase; RBF, renal blood flow; RPF, renal plasma flow; RVR, renal vascular resistance; SHR, spontaneously hypertensive rat; TGFβ, transforming growth factor β; UUO, unilateral ureteral obstruction; VEGF-A, vascular endothelial growth factor-A, ↑, increase; ↓, decrease.

**Table 2:** Animal studies using a GLP-1R agonist to treat non-diabetic kidney disease.

Animal Model	Renal Function	Kidney Biomarker	Reference
CIN (Sprague-Dawley rats)	↓BUN ↓Serum creatinine ↑Creatinine clearance ↓Proteinuria	↓Oxidative stress ↓Vascular dysfunction markers ↓Caspase-3 expression ↓Histopathological lesions	[56]
Ischemia/reperfusion AKI (Sprague-Dawley rats)	↓Serum creatinine	↓ATN score ↓Apoptosis ↓Macrophage infiltration ↑HO-1 expression	[57]
Cisplatin-induced AKI (C57BL/6 mice)	↓BUN ↓Serum creatinine	↓ATN score ↓Oxidative stress ↓Apoptosis	[58]
Nephrotoxic serum nephritis (C57BL/6J mice)	↓Albuminuria ↓Urinary NGAL	↓Glomerular crescents ↓Inflammation and fibrosis	[59]

Note: ATN, acute tubular necrosis; BUN, blood urea nitrogen; CIN, contrast-induced nephropathy; GLP-1R, glucagon-like peptide-1 receptor; HO-1, heme oxygenase-1; NGAL, neutrophil gelatinase-associated lipocalin, ↑, increase; ↓, decrease.

**Table 3:** Animal studies using DPP-4 inhibitors to treat non-diabetic kidney disease.

Animal Model	Renal Function	Kidney Biomarker	Reference
Ischemia/reperfusion AKI (Wistar-Han rats)	↓Serum creatinine	↓Tubular damage and inflammation ↓Apoptosis ↓Oxidative stress ↓CXCL10 mRNA	[67]
Ischemia/reperfusion AKI (Sprague-Dawley rats)	↓BUN ↓Serum creatinine ↓Proteinuria	↓Tubular injury ↓Oxidative stress ↓Pro-inflammatory markers ↓Apoptosis	[68]
Cisplatin-induced AKI (C57BL/6 mice)	↓BUN ↓Serum creatinine	↓ATN score ↓Oxidative stress ↓Apoptosis	[58]
Cisplatin-induced AKI (Sprague-Dawley rats)	↓BUN ↓Serum creatinine	↓Tubular injury ↓Interstitial fibrosis ↓Inflammation ↓Apoptosis ↑Proliferation of PTECs	[69]
UUO (C57BL/6J mice)	↔BUN ↔Serum creatinine	↓Interstitial fibrosis ↓Pro-inflammatory markers	[76]
UUO (C57BL/6J mice)	N/A	↓Interstitial fibrosis ↓Pro-fibrotic gene expression	[77]
5/6 nephrectomy (Wistar rats)	↓Albuminuria ↓Proteinuria	↓Interstitial fibrosis ↓Glomerular hypertrophy ↓Inflammation ↓Lipid peroxidation	[79]
5/6 nephrectomy (Sprague-Dawley rats)	↓BUN ↑Creatinine clearance	↓Glomerulosclerosis ↓Tubulointerstitial injury ↓PI3K-AKT activity ↓JNK phosphorylation ↓Apoptosis ↓Macrophage infiltration	[80]
Aging C57BL/6 mice	↓Serum creatinine ↓Cystatin C	↓Mesangial matrix ↓Interstitial fibrosis ↓Pro-inflammatory markers ↓Oxidative stress	[81]
Thy-1 glomerulonephritis (Sprague-Dawley rats)	↓Proteinuria	↓Glomerular injury ↓Macrophage infiltration	[82]
Anti-GBM nephritis (Wistar Kyoto rats)	↓Proteinuria	↓Glomerulosclerosis ↓Crescents ↓Tubular injury ↓Inflammation ↓Podocyte injury	[83]
Adriamycin nephropathy (BALB/c mice)	↓Proteinuria ↓Albuminuria	↓Macrophage infiltration ↑Podocyte number ↓Inflammation ↓Interstitial fibrosis	[84]
Doxorubicin nephropathy (Sprague-Dawley rats)	↔Proteinuria ↔Serum creatinine	↓Tubular injury ↓Interstitial fibrosis ↓Inflammatory cell infiltration ↓Oxidative stress	[85]
Hypertensive nephropathy (Dahl salt-sensitive rats)	↓Albuminuria ↓Serum creatinine	↓Interstitial fibrosis ↓Pro-inflammatory markers ↓Endothelial dysfunction ↓Oxidative stress	[87]
Hypertensive nephropathy (Dahl salt-sensitive rats)	↓Serum creatinine ↓Proteinuria ↓Albuminuria	↓Vascular injury ↓Pro-inflammatory gene expression ↓Pro-fibrotic gene expression	[88]

Note: AKI, acute kidney injury; ATN, acute tubular necrosis; CXCL10, C-X-C motif chemokine ligand 10; GBM, glomerular basement membrane; IRI, ischemia-reperfusion injury; JNK, c-Jun N-terminal kinase; N/A, not available; NHE3, sodium hydrogen exchanger type 3; PCR, protein to creatinine ratio; PI3K, phosphatidylinositol 3-kinase; PTECs, proximal tubular epithelial cells; UUO, unilateral ureteral obstruction; ↑, increase; ↓, decrease; ↔, no significant change.

**Table 4:** Animal studies using SGLT-2 inhibitors to treat non-diabetic kidney disease.

Animal Model	Renal Function	Kidney Biomarker	Reference
Ischemia/reperfusion AKI (C57BL/6 mice)	↓BUN ↓Serum creatinine	↓Tubular injury ↓Apoptosis ↑HIF-1	[112]
Ischemia/reperfusion AKI (C57BL/6J mice)	↔BUN ↔Creatinine clearance	↓Interstitial fibrosis ↓TGFβ mRNA	[113]
Cisplatin-induced AKI (C57BL/6 mice)	↓BUN ↓Serum creatinine	↓Tubular injury ↓Apoptosis ↓p53, p38, and JNK activity ↑AKT activation	[114]
5/6 nephrectomy (Sprague-Dawley rats)	↔BUN ↔Creatinine clearance	↔Glomerulosclerosis ↔Interstitial fibrosis	[117,118]
Oxalate nephropathy (C57BL/6N mice)	↔BUN ↔Serum creatinine	↔Oxalate crystal deposition ↔Pro-fibrotic gene expression	[119]
Adenine-induced CKD (Wistar rats)	↓BUN ↓Serum creatinine ↓Albuminuria	↓Oxidative markers ↑Nrf2 ↓Tubular necrosis and fibrosis	[120]
Adenine-induced CKD (C57BL/6JJcl mice)	↓Plasma creatinine ↔Proteinuria	↓Tubular dilatation ↓Interstitial fibrosis	[121]
Proteinuric nephropathy (C56BL/6N mice)	↓Proteinuria ↔GFR	↓Glomerular damage ↓Podocyte loss ↓Macrophage infiltration	[122]
Salt-sensitive hypertension (Sprague-Dawley rats)	↑Proteinuria ↑Creatinine clearance	↑HIF-1α, HO-1, and VEGF ↓Inflammatory markers ↓Oxidative stress	[124]
Ang II-induced hypertension (Sprague-Dawley rats)	↔GFR	↓Renal fibrosis ↓Type I and IV collagen expression ↓Inflammatory cell infiltration	[127]

Note: AKI, acute kidney injury; AKT, protein kinase B; Ang II, angiotensin II; BUN, blood urea nitrogen; CKD, chronic kidney disease; GFR, glomerular filtration rate; HIF-1, hypoxia-inducible factor-1; HO-1, heme oxygenase-1; JNK, c-Jun N-terminal kinase; Nrf2, nuclear factor erythroid 2-related factor 2; TGFβ, transforming growth factor β; VEGF, vascular endothelial growth factor; ↑, increase; ↓, decrease; ↔, no significant change.
